# Intralobar pulmonary sequestration in an adult: a case report

**DOI:** 10.1186/s13019-023-02127-2

**Published:** 2023-01-06

**Authors:** Prasanth Sadasivan Nair, Christopher Merry, Alexander White

**Affiliations:** Department of Cardiothoracic Surgery, The Alfred, 55 Commercial Road, Prahran, Melbourne, VIC 3004 Australia

**Keywords:** Pulmonary sequestration, Intralobar, Bronchopulmonary, Congenital lung anomaly

## Abstract

**Background:**

Pulmonary sequestration is a rare congenital lung anomaly, presenting mostly in childhood and adolescence.

**Case presentation:**

We report the case of a 26-year-old male patient presenting with pleuritic left sided chest pain and haemoptysis. Computed tomography of the chest showed features of intralobar pulmonary sequestration involving the left lower lobe, with arterial supply arising from the descending thoracic aorta above the diaphragm and normal venous drainage. Video assisted thoracic surgery was planned to perform a left lower lobectomy. Considering the risk of bleeding from the large artery supplying the sequestered segment, a posterolateral thoracotomy incision was made and left lower lobectomy was completed, with successful division of the arterial feeder. The patient was discharged home without complications. Pathologic examination of the specimen grossly revealed partial division of the lobe by two fissures with extensive adhesions into an upper and lower portion with no clear demarcation and a large vessel which enters the lower portion at the posterior inferior aspect, separate from the hilum with a diameter 10 mm. Microscopically, both portions of the lobe showed normally alveolated lung tissue with patchy recent intra-alveolar haemorrhage and evidence of chronic inflammation in the sequestered segment. There was no evidence of malignancy.

**Conclusion:**

This case highlights the rare presentation of pulmonary sequestration in adulthood and the importance of imaging to identify anomalous arterial supply to the sequestered segment in the left lower lobe of the lung. The use of safe surgical techniques to control the anomalous systemic arterial feeding vessel cannot be overemphasized.

## Background

Bronchopulmonary sequestration (BPS), simply referred to as pulmonary sequestration, is a rare congenital anomaly consisting of a non-functioning mass of lung tissue that lacks communication with the tracheobronchial tree. It has arterial supply from the systemic circulation and varying forms of venous drainage [1]. The various subtypes include: Intralobar sequestration (ILS), Extralobar sequestration (ELS), Hybrid BPS/congenital pulmonary airway malformation (CPAM) lesions and bronchopulmonary foregut malformation. In general, congenital abnormalities of the lower respiratory tract are rare, found in approximately 1 in 10,000 to 35,000 live births [2]. Among these, the most common CPAM, while BPS represents only 0.15 to 6.40 percent [3]. ILS is overall the most common form, comprising approximately 75 to 90 percent of sequestrations, while 10 to 25 percent are ELS [3, 4]. ILS affects both sexes equally, while ELS is predominant in males in most [1, 5], but not all [6], reports. In a series of ELS cases diagnosed antenatally, the ratio of males to females was three to one [7].

Chronic respiratory infection is the most common mode of presentation, although sequestrations may be discovered incidentally on radiographic studies. In ILS, anomalous systemic arterial supply is via the DTA (72%), as seen in our case, via abdominal aorta, celiac axis, or splenic artery (21%), via intercostal artery (3%), and rarely via the subclavian, internal thoracic, and pericardiacophrenic arteries. Venous drainage is usually via the pulmonary veins, but it can also occur through the azygos vein/hemiazygos system, portal vein, right atrium, or inferior vena cava (IVC) [8, 9].

## Case presentation

A 26-year-old Caucasian male presented with history of intermittent episodes of pleuritic chest associated with three episodes of haemoptysis, three days prior to admission. The volume of blood coughed out was 15 ml in each instance and the chest pain lasted only a few minutes, resolving spontaneously. He denied any fever, shortness of breath, palpitation, loss of appetite or weight loss. His past medical history was significant for recurrent pneumonia and epididymitis during childhood and adolescence. He previously underwent scrotal exploration and orchidopexy for torsion of the right testis 9 months ago. He denied history of substance abuse or sexually transmitted diseases. Physical examination revealed that his vital signs were within normal limits, chest was clear with no added sounds and there was no calf tenderness.

**Fig. 1:**
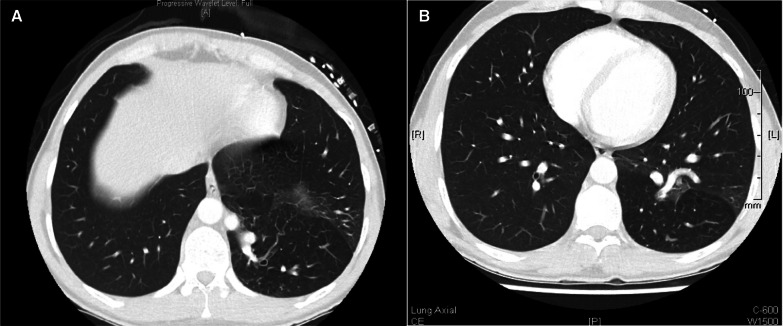
**A**, **B** CT scan showing aberrant formation of segmental lung tissue within the left lower lobe

**Fig. 2:**
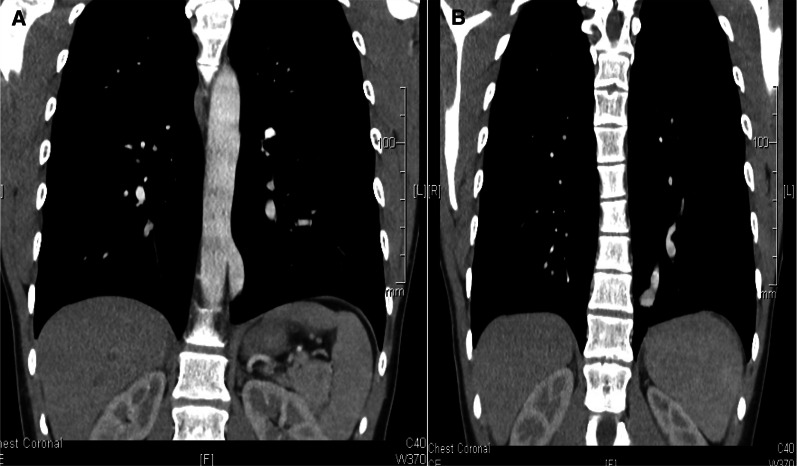
**A**, **B** Coronal CT views showing systemic arterial supply taking origin from the DTA

Routine blood tests including a D-dimer level and COVID-19 PCR test did not reveal any abnormalities. Chest X-ray showed normal cardio-mediastinal contour and no evidence of any lung lesions. Further investigation with a CT chest showed aberrant formation of segmental lung tissue within the left lower lobe with air trapping in a postero-basal location. There was no discernable communication to the bronchial tree (Fig. 1A, B). This region had a systemic arterial supply taking origin from the descending thoracic aorta above the diaphragm (Fig. 2A, B). Venous drainage was via the pulmonary venous system, namely the left inferior pulmonary vein. There was also ground glass change within the sequestered segment which was suggestive of infection and/or haemorrhage. Findings were suggestive of an intra-lobar pulmonary sequestration. A lung function test performed showed normal spirometry values and CO transfer factor.

**Fig. 3:**
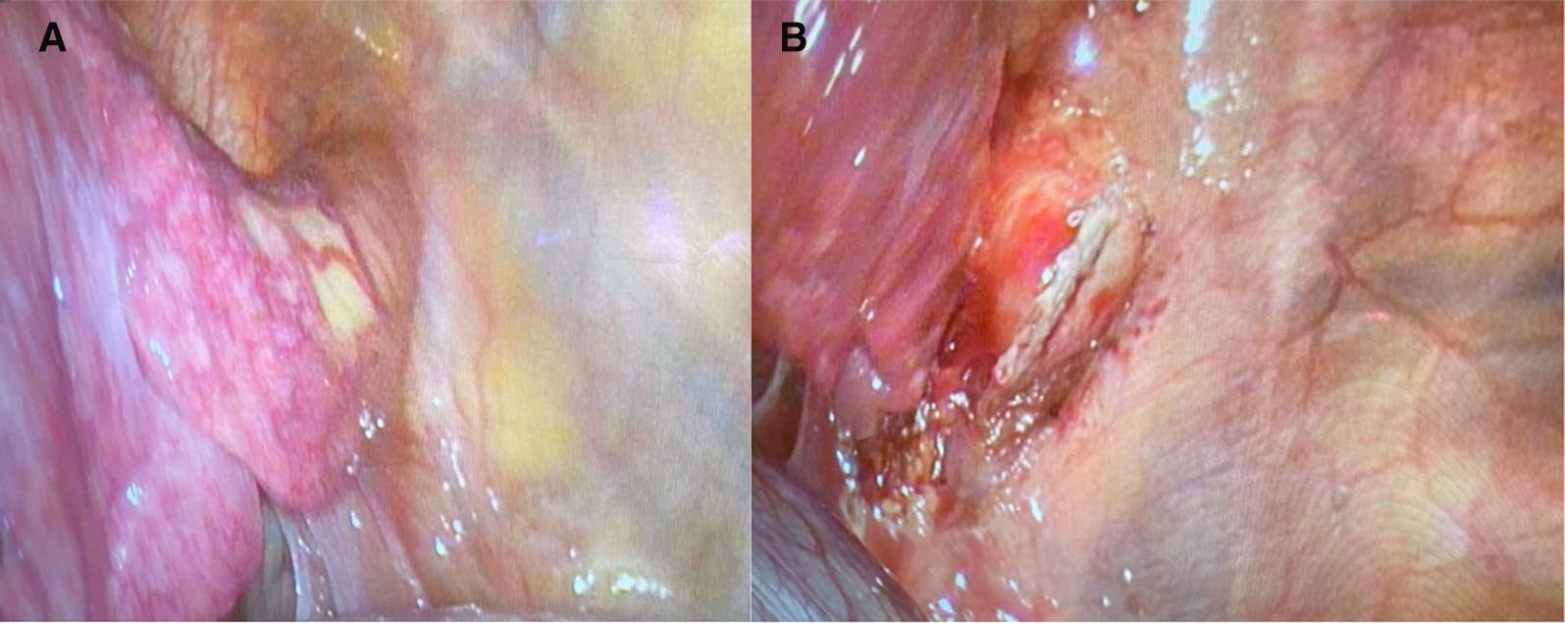
**A**, **B** Intra-operative photographs of the large anomalous artery arising from the DTA which was subsequently stapled and divided

 There were no further episodes of haemoptysis following admission and the case was referred to us for surgical management. The patient was positioned in the right lateral decubitus position after induction of anaesthaesia and double lumen endotracheal tube was inserted to isolate the left lung. Initially, a VATS port was introduced in the 8^th^ intercostal space in the anterior axillary line and the pleural cavity was inspected. An anomalous artery approximately 3 cm in diameter was seen arising from the lower part of the descending thoracic aorta supplying the sequestered segment (Fig. 3A). The venous drainage was to the inferior pulmonary vein and there was no separate airway communication of the sequestered lung to the tracheobronchial tree.

In order to achieve safe control and division of the large anomalous artery, it was decided to make a separate posterolateral thoracotomy incision (about 6 cm in length). The fifth intercostal space was entered after splitting the latissimus dorsi muscle and sparing the serratus anterior. The anomalous artery was stapled and divided using an Endo GIA™ 45 mm vascular stapler (Fig. 3B). The inferior pulmonary vein, the ongoing lower lobe pulmonary artery and the superior segmental artery were stapled and divided using endo GIA staplers. The left lower lobe bronchus was divided after test inflation of the lung and the left lower lobe was delivered. Warm saline was infiltrated and inflation of the lung showed no air leak. An indwelling extrapleural catheter was inserted under direct vision for pain relief. The wound was closed in layers with a 24 Fr drain placed through the port site. The patient was extubated in theatre and shifted to recovery in a stable state. Post-operative Chest X-ray showed good expansion of the left lung. The pleural drain was removed on post-operative day 2 and the patient was discharged home uneventfully on day 3.

Histopathology of the specimen grossly demonstrated a subpleural focally cystic area with dense haemorrhagic parenchyma measuring 20 × 10 × 15 mm in the superior segment. Microscopy showed the artery entering the lower segment and patchy recent intra-alveolar haemorrhage in normally alveolated lung. There were also foci of lymphocytic interstitial inflammation.

The patient had an uncomplicated post-operative course and was asymptomatic when reviewed after 6 weeks from surgery.

## Discussion

The pathogenesis of BPS is poorly understood. The most widely accepted embryologic theory is that BPS originates early in the pseudo-glandular stage of lung development (5 to 17 weeks of gestation), prior to separation of the aortic and pulmonary circulations [10]. This would explain the wide spectrum of pathology observed, including the connections to the systemic circulation, the presence of separate visceral pleura in ELS or lack thereof in ILS, the occurrence of hybrid lesions with features of BPS and CPAM, and the occasional associations with bronchogenic cysts or connections to the foregut, as well as associated anomalies such as congenital diaphragmatic hernia [10–12]. ILS is four times more common than ELS. ILS presents late in childhood or adolescence with recurrent pulmonary infection while ELS more commonly presents in newborns with respiratory distress, cyanosis, and infection. The most common location of ILS is in the posterior basal segments of the lung and nearly two third appearing in the left lung. Associated congenital anomalies are uncommon in ILS [13].

Mechanical separation from the rest of the organ by compression from vascular structures, traction by aberrant systemic vessels, or inadequate pulmonary blood flow is another postulate. However, this does not completely explain all types of lesions, specifically bronchopulmonary foregut malformation [14].

Clinical manifestation of BPS is variable and depends upon the type, size, and location of the lesion. Most cases are asymptomatic. If symptomatic, BPS usually presents with respiratory distress in the neonatal period. ILS or hybrid forms often present in adolescence or adulthood, with infection [15]. ELS is less likely to get infected and is diagnosed incidentally on imaging. The differential diagnoses of pulmonary sequestration include persistent pneumonia, lung abscess, congenital pulmonary airway malformation, bronchogenic cyst, pulmonary arteriovenous malformation and Scimitar syndrome. Symptomatic patients are ideally treated with surgical excision which is curative and associated with minimal morbidity [16]. The options include lobectomy or segmental resection with or without preoperative embolization of feeding vessels. In any case, CT chest (additional 3D reconstructed images of the anomalous arteries can be helpful, although not a necessity) or Magnetic Resonance Imaging (MRI) must be performed beforehand to confirm the diagnosis, identify the anomalous arterial supply and venous drainage, and assist surgical planning.

Surgical resection is advocated for most of these lesions because of the likelihood of recurrent infection and the possibility of haemorrhage and ILS often requires lobectomy. Although thoracoscopic resection has been reported with low morbidity and mortality [17], open thoracotomy for safe isolation and division of the anomalous systemic feeding arteries is recommended to prevent complications.

## Conclusion

ILS is a rare congenital anomaly that presents with recurrent pulmonary infections in late childhood or adolescence. Accurate identification of anomalous systemic arterial feeding vessel/s on imaging helps in pre-operative planning. We advocate open thoracotomy for safe isolation and division of the anomalous feeding arterial vessel.

## Data Availability

Not applicable.

## Abbreviations

CT Chest: Computed tomography of the chest
DTA: Descending thoracic aorta
VATS: Video assisted thoracic surgery
BPS: Bronchopulmonary sequestration
ILS: Intralobar sequestration
ELS: Extralobar sequestration
CPAM: Congenital pulmonary airway malformation
IVC: Inferior vena cava
